# Cardiac toxicity and intervention strategies during thoracic cancer radiotherapy

**DOI:** 10.3389/fonc.2025.1638035

**Published:** 2025-08-04

**Authors:** Youjin Kong, Chao Yang, Lingling Xie, Yan Zhao, Zheng Rong, Xinbin Zhou, Wei Mao

**Affiliations:** ^1^ Department of Cardiology, The First Affiliated Hospital of Zhejiang Chinese Medical University (Zhejiang Provincial Hospital of Chinese Medicine), Hangzhou, Zhejiang, China; ^2^ The Fourth School of Clinical Medicine, Zhejiang Chinese Medical University, Hangzhou, Zhejiang, China; ^3^ Zhejiang Key Laboratory of Integrative Chinese and Western Medicine for Diagnosis and Treatment of Circulatory Diseases, Zhejiang Hospital (Affiliated Zhejiang Hospital, Zhejiang University School of Medicine), Hangzhou, Zhejiang, China; ^4^ Department of Cardiology, Zhejiang Hospital (Affiliated Zhejiang Hospital, Zhejiang University School of Medicine), Hangzhou, Zhejiang, China

**Keywords:** thoracic cancer, radiotherapy, RIHD, cardiotoxicity, cardiac fibrosis, intervention, radiation-induced heart disease

## Abstract

Radiation-induced heart disease (RIHD) represents a major dose-limiting complication of thoracic radiotherapy, with a multifaceted pathogenesis involving endothelial dysfunction, chronic oxidative stress, and progressive fibrotic remodeling. Emerging evidence reveals distinct molecular mechanisms underlying RIHD’s heterogeneous clinical manifestations, including pericarditis, accelerated coronary artery disease, cardiomyopathy, valvular degeneration, and conduction abnormalities—which often manifest after prolonged latency periods. Modern radiotherapy techniques have reduced but not eliminated cardiac toxicity, particularly in high-risk populations. Advanced imaging modalities and biomarkers now enable earlier detection, though diagnostic challenges persist. While current management remains largely extrapolated from conventional cardiovascular therapies, novel targeted interventions show preclinical promise. This review synthesizes contemporary understanding of RIHD pathophysiology, risk stratification paradigms, and evolving cardioprotective strategies, while highlighting critical knowledge gaps requiring translational investigation to optimize outcomes for cancer survivors.

## 1 Introduction

Radiation-induced heart disease (RIHD) has become an increasingly significant clinical challenge, particularly among cancer survivors who have undergone thoracic radiotherapy (1). As a fundamental component in the multidisciplinary treatment of thoracic malignancies, including breast cancer, lung cancer, esophageal cancer, and mediastinal lymphomas (2, 3), radiotherapy has contributed to improved survival outcomes through advancements in precision techniques and multimodal therapeutic strategies (4). However, these therapeutic benefits are accompanied by the risk of both acute and delayed toxicities to surrounding normal tissues, with radiation-induced cardiac injury representing one of the most severe and potentially life-threatening complications (5–7). RIHD encompasses a spectrum of cardiovascular pathologies whose clinical manifestations and severity are influenced by several critical factors: the specific cardiac substructures within the radiation field, dose-volume parameters, and treatment field design. The disease spectrum includes cardiomyopathy, pericardial disease, accelerated coronary artery atherosclerosis, valvular dysfunction, and conduction system abnormalities. Clinical presentations vary widely, from overt symptomatic disease to subclinical dysfunction detectable only through advanced imaging modalities or sensitive biomarker analysis (8–10).

The pathogenesis of RIHD is complex, involving acute microvascular endothelial injury, inflammatory responses, and chronic progressive fibrotic processes (11). At the molecular level, radiation induces DNA damage and activates signaling pathways such as TGF-β/Smad, leading to sustained pro-fibrotic responses (12). Despite improvements in radiotherapy delivery techniques, RIHD remains a major dose-limiting factor, particularly in high-risk populations such as those with pre-existing cardiovascular comorbidities or those receiving cardiotoxic chemotherapy (13). Characterized by a prolonged latency period (typically 5–10 years post-exposure) and heterogeneous clinical presentations, RIHD poses substantial challenges for early detection and intervention (14). This review synthesizes current mechanistic understanding of both acute and chronic RIHD phases, elucidates the pivotal role of molecular pathways such as TGF-β/Smad signaling, and evaluates emerging diagnostic and therapeutic approaches, including proton therapy, FLASH irradiation, and novel anti-fibrotic agents. A deeper understanding of the mechanisms and risk factors of RIHD is essential for developing individualized treatment strategies that balance cancer control and cardio-protection.

## 2 Pathophysiological mechanisms of RIHD

### 2.1 Acute and chronic RIHD

Acute RIHD develops rapidly, emerging within minutes to hours following radiation exposure, and is primarily mediated by neutrophil infiltration into myocardial tissue (15). These neutrophils trigger a potent inflammatory cascade by recruiting macrophages and other immune cells, which subsequently release key pro-inflammatory mediators including tumor necrosis factor, interleukin-1, interleukin-6, monocyte chemoattractant protein-1, platelet-derived growth factor, and transforming growth factor-beta (16). This cytokine storm exacerbates acute tissue injury, establishing acute RIHD as an inflammation-driven pathological process (17). In contrast, chronic RIHD develops through prolonged oxidative stress and reactive oxygen species accumulation. Inflammatory cell infiltration plays a critical role in perpetuating oxidative damage and pathological cardiac remodeling (18). Persistent oxidative stress and cytokine dysregulation induce myocardial fibrosis and hypertrophy, compromising cardiac function and microvascular perfusion. Progressive vascular occlusion from cumulative radiation exposure leads to ischemic cardiomyocyte necrosis, while excessive collagen and extracellular matrix deposition drive fibrotic degeneration, resulting in irreversible myocardial damage (19–21).

### 2.2 TGF-β/Smad signaling in radiation-induced heart disease

The pathogenesis of RIHD is strongly linked to the activation of pro-fibrotic signaling cascades, with the TGF-β/Smad pathway being a key mediator (22, 23). Following ionizing radiation exposure, latent TGF-β, stored in the extracellular matrix, undergoes activation via ROS-dependent mechanisms and proteolytic cleavage (24). Once activated, TGF-β binds to its receptors (TGFBR1/TGFBR2) on endothelial cells and cardiac fibroblasts, triggering the phosphorylation of Smad2/3 (25). These phosphorylated Smads form a complex with Smad4 and translocate to the nucleus, where they induce the transcription of fibrosis-related genes, including PAI-1, COL3A1, and COL1A1 (26, 27). Beyond promoting EMT and myofibroblast differentiation, the TGF-β/Smad pathway exacerbates tissue stiffening (28, 29). Additionally, its crosstalk with NF-κB signaling and p38 MAPK further amplifies fibrotic progression. Chronic TGF-β/Smad activation sustains adverse myocardial remodeling, microvascular loss, and inflammation, hallmarks of advanced RIHD (9).

## 3 Clinical spectrum of radiation-induced cardiac injuries

### 3.1 Radiation pericarditis

RIHD affects multiple cardiac structures, including the pericardium, myocardium, coronary arteries, valves, and conduction system, either independently or concurrently (30, 31). The onset of clinical manifestations varies from weeks to decades following radiotherapy, influenced by radiation dose and anatomical targeting (4). Pericardial involvement is particularly common, predominantly due to microvascular endothelial injury and subsequent fibrotic changes (32). The condition encompasses constrictive pericarditis, chronic pericarditis, and acute radiation pericarditis. Acute pericarditis is rare, typically emerging during or immediately after radiation exposure, characterized by fever, pleuritic chest pain, electrocardiographic alterations, and mild biomarker elevation. While most cases are self-limiting or manageable with NSAIDs and diuretics, a subset may progress to chronic inflammation, necessitating long-term monitoring (33). Chronic pericarditis frequently develops within 12 months post-radiation, commonly presenting as pericardial effusion. Research indicates a median onset of 5.3 months, with a strong dose-dependent association, pericardial V30 >46% correlates with a 73% effusion incidence compared to 13% at V30 <46% (34, 35). Sustained inflammation may cause pericardial fibrosis, compromised diastolic filling, and life-threatening tamponade, occasionally requiring pericardiocentesis or surgical intervention. Echocardiography serves as the primary diagnostic modality (36–38). Pathological thickening (exceeding 17 mm) restricts ventricular filling, leading to progressive heart failure within a decade. Although NSAIDs can provide symptomatic relief in mild cases, advanced disease with significant hemodynamic compromise necessitates invasive interventions (39).

### 3.2 Radiation-induced coronary artery disease

**Figure 1:**
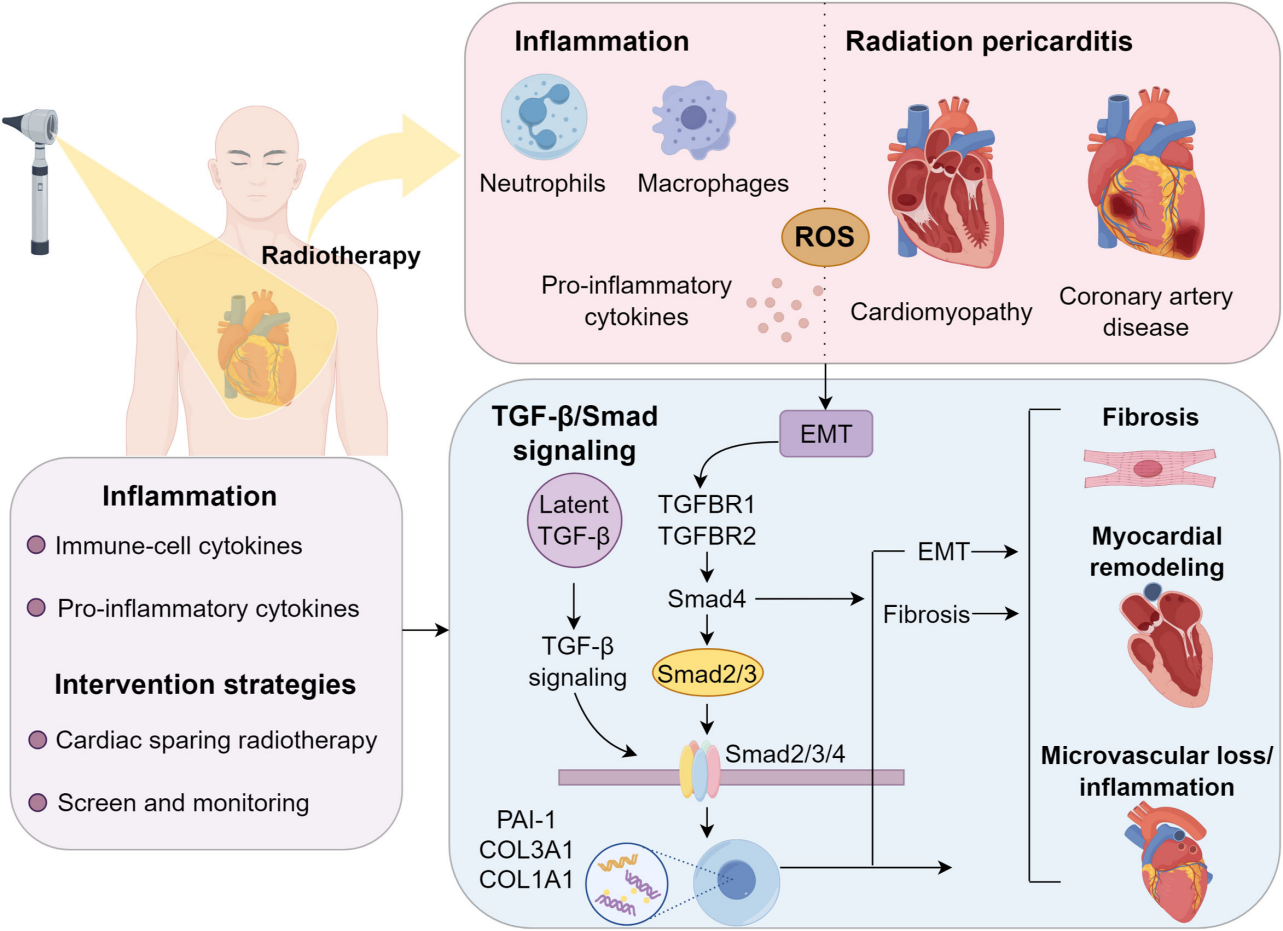
Radiation-induced cardiac toxicity in thoracic oncology.

Coronary artery injury is a pivotal contributor to the elevated incidence of cardiovascular morbidity post-radiotherapy (40). The clinical presentation includes angina, dyspnea, heart failure, syncope, and, in rare cases, sudden cardiac death (41, 42). Radiation accelerates atherosclerosis through endothelial dysfunction, leading to plaque formation. Notably, the left anterior descending artery, frequently within the high-dose volume during left-sided breast cancer radiotherapy, is particularly vulnerable (43, 44). Radiation triggers vascular inflammation, microvascular impairment, and subendothelial fibrosis, predisposing to unstable plaque development, especially at arterial bifurcations. Concurrent cardiovascular risk factors, such as hyperlipidemia, exacerbate disease progression. Tjessem et al. highlighted a synergistic effect between radiation exposure and hypercholesterolemia in accelerating coronary artery disease (45). Long-term survivors face an escalating risk of RICAD with advancing age, further amplified by comorbidities like ischemic heart disease (IHD), diabetes, dyslipidemia, smoking, and chronic obstructive pulmonary disease (COPD) (46). Therapeutic approaches for RICAD mirror those for conventional coronary artery disease, including pharmacotherapy and revascularization via percutaneous coronary intervention (PCI). However, post-radiotherapy patients demonstrate higher rates of graft restenosis following coronary artery bypass grafting (CABG), necessitating careful long-term surveillance (39) (**Figure 1**).

### 3.3 Radiation-induced myocardial injury

Radiotherapy-induced direct myocardial injury and endothelial dysfunction promote intravascular collagen accumulation, causing capillary constriction, myocardial ischemia, and subsequent fibrotic tissue remodeling (47). These pathological changes progressively compromise both systolic and diastolic cardiac performance, ultimately leading to heart failure (48, 49). Radiation-induced myocardial injury typically follows an indolent clinical course, remaining subclinical for more than a decade and consequently leading to significant underdiagnosis, with reported clinical detection rates as low as 10% (50). Symptomatic manifestations commonly include reduced exercise capacity and diminished left ventricular ejection fraction (LVEF), which may also show mild reduction during resting conditions. Mediastinal irradiation in Hodgkin lymphoma survivors is associated with increased risk of congestive heart failure compared to non-irradiated populations. Retrospective cohort analyses reveal a cumulative 5-year CHF incidence of 5.9% (95% CI 3.4-9.6) following radiotherapy, with cardiac radiation doses exceeding 15 Gy conferring higher risk for major cardiovascular complications including heart failure, myocardial infarction, and valvular disease (51). Therapeutic strategies mirror standard cardiomyopathy management, incorporating ACE inhibitors, ARBs, aldosterone antagonists, and β-blockers.

### 3.4 Radiation-induced valvular heart disease

Radiation exposure significantly compromises cardiac valve integrity, inducing pathological changes such as thickening, calcification, and fibrosis, which progress from valvular regurgitation to stenosis. Studies indicate that valvular abnormalities occur in up to 81% of RIHD patients, with clinically significant dysfunction observed in 6% of cases (52). The aortic and mitral valves demonstrate the highest susceptibility, primarily due to their anatomical position within radiation fields and exposure to elevated pressure gradients (53). Histopathological analysis reveals chronic endothelial injury and collagen accumulation as central features, with radiation-induced activation of fibroblasts and valvular interstitial cells contributing to fibrotic remodeling (54). Myxoid degeneration and calcific nodules are frequently observed in irradiated valves. Moreover, TGF-β signaling plays a pivotal role in promoting valvular fibrosis post-radiation (22, 55). Clinically, radiation-induced valvular disease (RIVD) can remain asymptomatic for years, underscoring the importance of routine long-term monitoring (56). Mediastinal radiation therapy (MRT) significantly elevates the risk of valvular heart disease, with a strong dose-dependent relationship particularly evident at radiation doses exceeding 30 Gy. The aortic valve demonstrates particular vulnerability, showing the highest incidence of radiation-induced damage (57). These valvular abnormalities typically manifest after a prolonged latency period, with their prevalence showing a progressive increase over time. Long-term follow-up studies demonstrate that approximately 31% of Hodgkin lymphoma survivors develop valvular regurgitation within 10 years post-MRT, with this proportion exceeding 90% after 22 years (58). When compared to non-irradiated HL survivors, MRT recipients not only face substantially higher risks of valvular dysfunction but also require surgical intervention more frequently (59–63). For severe aortic stenosis cases, surgical aortic valve replacement remains the gold standard treatment, while transcatheter aortic valve replacement (TAVR) has emerged as an effective alternative for patients with elevated surgical risk (64).

### 3.5 Radiation-induced cardiac conduction abnormalities

Radiation-induced conduction system disturbances, though uncommon, may arise from myocardial fibrosis, localized ischemia, or direct injury to the sinoatrial or atrioventricular nodes (49, 50). Clinically, these disturbances manifest as atrioventricular block, sick sinus syndrome, QTc prolongation, and supraventricular or ventricular arrhythmias. Right bundle branch block is particularly prevalent due to the anatomical proximity of the conduction bundle to the irradiated endocardial surface (51). The underlying mechanisms involve radiation-induced dysregulation of ion channel expression and electrophysiological remodeling, driven by chronic inflammation and fibrotic infiltration into conduction pathways. Pediatric and adolescent cancer survivors are at heightened risk, with conduction abnormalities often emerging decades after radiation exposure. Studies report that 12.5% of irradiated pediatric survivors exhibit a resting QTc interval ≥0.44 seconds (52), while Hodgkin lymphoma survivors face a twofold increased risk of requiring pacemaker or implantable cardioverter-defibrillator (ICD) implantation compared to the general population (53). Although asymptomatic cases typically require no intervention, symptomatic patients may benefit from pacemaker implantation or radiofrequency ablation. A systematic classification of these radiation-associated cardiac complications facilitates early detection and personalized management strategies (**Supplementary Table S1**).

## 4 Modifiable risk factors and dose-response relationships

he development of radiation-induced heart disease (RIHD) is modulated by three key modifiable factors: radiation dose, chemotherapy regimen, and cardiac exposure volume. In Hodgkin lymphoma, radiation demonstrates a clear dose-dependent association with valvular pathology, where higher doses progressively exacerbate valvular dysfunction (57). Similarly, in breast cancer radiotherapy, left-sided irradiation confers greater cardiac toxicity than right-sided treatment due to increased cardiac exposure, leading to higher RIHD incidence (65). Concurrent chemotherapy further amplifies radiation-associated cardiotoxicity. Anthracycline-based regimens are particularly detrimental, with patients receiving mediastinal radiotherapy plus anthracyclines exhibiting twice the incidence of valvular abnormalities compared to non-anthracycline protocols (66). Moreover, valvular disease severity correlates positively with cumulative anthracycline dose, underscoring its compounding effect on cardiac damage (59). Detailed dose-response analyses in HL patients reveal critical thresholds for cardiac substructure irradiation. Significant valvular pathology occurs when >63% of the left atrium receives ≥25 Gy or >25% of the left ventricle receives ≥30 Gy (67). Furthermore, whole-heart irradiation exceeding 33 Gy markedly elevates valvular disease risk, establishing this as a critical dose threshold.

## 5 Multimodality diagnostic approaches

Diagnosing radiation-induced heart disease (RIHD) presents a clinical challenge due to its often insidious and delayed presentation (68). As a diagnosis of exclusion, it necessitates a comprehensive clinical evaluation, particularly in patients with a history of thoracic radiotherapy. Echocardiography remains the cornerstone of diagnostic evaluation, enabling the detection of subclinical cardiac dysfunction even in the early post-radiation period (69, 70). Three-dimensional echocardiography and contrast-enhanced techniques were recommended to improve the accuracy of left ventricular ejection fraction (LVEF) quantification (71). Current guidelines recommend baseline echocardiography before radiotherapy, repeated assessments during and three months post-treatment, with subsequent periodic LVEF monitoring. For asymptomatic patients, follow-up echocardiograms every five years are advised (72). Cardiac magnetic resonance imaging (cMRI) remains the reference standard for evaluating cardiac anatomy, function, and perfusion, while also providing critical insights into pericardial and coronary pathology (73, 74). Additionally, myocardial biomarkers, particularly cardiac troponins (TnI and TnT), are pivotal in detecting myocardial injury, with troponin T (TnT) being the most clinically relevant in radiation-induced cardiac damage (75). High-sensitivity troponin T (hs-cTnT) further enhances early detection of minimal myocardial injury during radiotherapy (76, 77). However, the predictive value of these biomarkers in routine clinical practice remains limited by several factors, including baseline variability among patients, interference from non-radiation-related comorbidities (78, 79). Furthermore, the sensitivity and specificity of these markers in differentiating radiation-induced damage from other cardiotoxic insults, such as chemotherapy, are still under evaluation (80). Therefore, while hs-cTnT and NT-proBNP show potential for early RIHD detection, their clinical application should be integrated with imaging and risk stratification tools rather than used in isolation.

## 6 Cardioprotective strategies in radiotherapy

### 6.1 Optimizing radiotherapy protocols

To mitigate RIHD incidence, strategic modifications in radiation dose parameters and cardiac-sparing techniques must be complemented by vigilant post-treatment surveillance and proactive interventions. Minimizing cardiac radiation exposure remains the cornerstone of RIHD prevention. Current approaches include risk-adapted personalized planning with dose/fractionation adjustments, image-guided field reduction, respiratory gating, and advanced modalities like intensity-modulated radiotherapy (IMRT) or proton beam therapy (81–83). Given the dose-dependent cardiotoxicity, contemporary protocols advocate limiting daily doses to ≤2 Gy. A phase IIb randomized trial (n=145) in locally advanced esophageal cancer demonstrated proton therapy’s superiority over IMRT in reducing composite toxicity while preserving oncological outcomes (84). Beyond clinical techniques, several preclinical innovations show promise. FLASH radiotherapy, an innovative approach delivering millisecond ultra-high dose rates, has cardioprotective potential in animal models, showing significantly attenuated cardiac fibrosis, inflammatory responses, and oxidative damage without compromising tumor control (85–88). However, its translation into clinical practice awaits further validation.

### 6.2 Screening and surveillance of RIHD

Patients receiving thoracic radiotherapy or irradiation near the heart require lifelong systematic monitoring. Current clinical guidelines recommend a comprehensive baseline assessment, including physical examination and transthoracic echocardiography, before initiating radiation therapy (89). Echocardiography serves as the primary surveillance tool for RIHD due to its widespread availability, cost efficiency, and capacity to evaluate left ventricular ejection fraction (LVEF), diastolic function, and pericardial effusion (2, 71). Nevertheless, its diagnostic accuracy for early myocardial fibrosis or regional wall motion abnormalities remains suboptimal, particularly in cases with poor acoustic windows (90, 91). Advanced imaging modalities such as cMRI are clinically used when echocardiographic findings are inconclusive, providing superior tissue characterization through late gadolinium enhancement and mapping techniques (92, 93). Although cMRI represents the reference standard for myocardial fibrosis assessment, its clinical application may be restricted by limited accessibility, high costs, and contraindications such as implanted devices (90). Multidetector computed tomography (MDCT) offers high-resolution visualization of coronary artery calcification and early atherosclerotic changes, particularly valuable for radiation-induced coronary artery disease (RICAD) evaluation, yet it provides no functional data and involves radiation exposure (94). Nuclear myocardial perfusion imaging detects ischemic regions but suffers from inferior spatial resolution and potential attenuation artifacts, with inconsistent diagnostic performance (95). Therefore, an integrated diagnostic approach—grounded in baseline risk stratification and long-term monitoring—is critical for early RIHD identification, enabling prompt cardioprotective measures and personalized management in cancer survivors. The predictive value of cardiac biomarkers for radiation-related cardiovascular toxicity remains under investigation. Although elevated troponin and NT-proBNP levels have been observed in patients undergoing radiotherapy, their clinical utility as early diagnostic tools for RIHD requires further large-scale validation (96, 97).

### 6.3 Treatment of RIHD

Several therapeutic strategies show potential for addressing radiation-induced cardiovascular injury. Among clinically recommended therapies, ACE inhibitors and β-blockers have been adopted for managing radiation-related cardiomyopathy, mirroring standard heart failure protocols (98, 99). However, their prophylactic use in preventing RIHD is still under investigation. In preclinical studies, captopril has demonstrated efficacy in reducing cardiac damage post-irradiation, suggesting potential for cardioprotection (100). Statins, commonly used in clinical settings for dyslipidemia, may also attenuate radiation-induced inflammation and fibrosis based on animal model data, though clinical evidence remains limited (101). Additionally, interleukin-1 blockade using agents like anakinra has been investigated for mitigating radiation-associated vascular inflammation (102). Despite these promising findings, none of these interventions have been widely adopted in clinical practice due to insufficient evidence. Further validation through large-scale randomized trials is necessary to establish their efficacy and safety.

## 7 Conclusion

Radiation-induced heart disease (RIHD) remains a major late complication of thoracic radiotherapy, driven by endothelial injury, chronic inflammation, and fibrotic remodeling. Its clinical manifestations include pericardial disease, coronary artery disease, cardiomyopathy, valvular dysfunction, and conduction abnormalities, often appearing years after treatment. Although modern radiotherapy techniques such as intensity-modulated radiotherapy, proton therapy, and FLASH irradiation have reduced cardiac exposure, the risk remains, especially in patients with pre-existing cardiovascular conditions or those receiving cardiotoxic chemotherapy. Diagnosis is challenging due to the delayed and subtle onset of RIHD, requiring a multimodal strategy involving echocardiography, cardiac magnetic resonance imaging, and cardiac biomarkers. Current treatments largely follow standard cardiovascular management, while emerging approaches including angiotensin-converting enzyme inhibitors, statins, and interleukin-1 blockade show promise in preclinical models but need further clinical validation.

Critical research gaps persist in the prevention and management of RIHD. There is an urgent need for long-term prospective studies assessing cardiovascular outcomes in cancer survivors, as well as the development of validated risk prediction models tailored to cancer type, radiation dose, and treatment strategy. Translational efforts should focus on identifying molecular drivers of RIHD and advancing targeted antifibrotic and anti-inflammatory therapies. Future research priorities include biomarker-guided surveillance protocols, longitudinal outcome registries, and clinical trials evaluating cardioprotective agents that target pathways such as transforming growth factor beta and chronic inflammation. Incorporating cardio-oncology principles into survivorship care, including routine cardiovascular screening and multidisciplinary collaboration, is essential to improving long-term patient outcomes.

## References

[1] Wang KX Ye C Yang X Ma P Yan C Luo L. New insights into the understanding of mechanisms of radiation-induced heart disease. Curr Treat Options Oncol. (2023) 24:12–29. doi: 10.1007/s11864-022-01041-4, PMID: 36598620

[2] Quintero-Martinez JA Cordova-Madera SN Villarraga HR. Radiation-induced heart disease. J Clin Med. (2021) 11:146. doi: 10.3390/jcm11010146, PMID: 35011887 PMC8745750

[3] Khalifa J Lerouge D Le Péchoux C Pourel N Darréon J Mornex F. Radiotherapy for primary lung cancer. Cancer Radiother. (2022) 26:231–43. doi: 10.1016/j.canrad.2021.11.005, PMID: 34953709

[4] Mitchell JD Cehic DA Morgia M Bergom C Toohey J Guerrero PA. Cardiovascular manifestations from therapeutic radiation: A multidisciplinary expert consensus statement from the international cardio-oncology society. JACC Cardio Oncol. (2021) 3:360–80. doi: 10.1016/j.jaccao.2021.06.003, PMID: 34604797 PMC8463721

[5] Pedersen LN Schiffer W Mitchell JD Bergom C. Radiation-induced cardiac dysfunction: Practical implications. Kardiol Pol. (2022) 80:256–65. doi: 10.33963/KP.a2022.0066, PMID: 35238396 PMC13234929

[6] de Groot C Beukema JC Langendijk JA van der Laan HP van Luijk P van Melle JP. Radiation-induced myocardial fibrosis in long-term esophageal cancer survivors. Int J Radiat Oncol Biol Phys. (2021) 110:1013–21. doi: 10.1016/j.ijrobp.2021.02.007, PMID: 33577960

[7] Mrotzek SM Rassaf T Totzeck M. Cardiovascular damage associated with chest irradiation. Front Cardiovasc Med. (2020) 7:41. doi: 10.3389/fcvm.2020.00041, PMID: 32266294 PMC7103638

[8] Lee ChuyKNahhasODominicPLopezCTonorezosESidlowR. Cardiovascular complications associated with mediastinal radiation. Curr Treat Options Cardiovasc Med. (2019) 21:31. doi: 10.1007/s11936-019-0737-0, PMID: 31161453

[9] Wang H Wei J Zheng Q Meng L Xin Y Yin X. Radiation-induced heart disease: a review of classification, mechanism and prevention. Int J Biol Sci. (2019) 15:2128–38. doi: 10.7150/ijbs.35460, PMID: 31592122 PMC6775290

[10] Patel D Parikh C Gharavi D Patil S Werner T Simone CB 2nd. Radiation-Induced coronary artery disease in lung and breast cancer patients: insights from PET imaging and long-term risk assessment. PET Clin. (2025) 20:231–41. doi: 10.1016/j.cpet.2025.01.006, PMID: 39955159

[11] Gawali B Sridharan V Krager KJ Boerma M Pawar SA. TLR4-A pertinent player in radiation-induced heart disease? Genes (Basel). (2023) 14:1002. doi: 10.3390/genes14051002, PMID: 37239362 PMC10218711

[12] Wu B Zhao S Zhang J Liu Y Bai J Wang G. PD-1 inhibitor aggravate irradiation-induced myocardial fibrosis by regulating TGF-β1/smads signaling pathway via GSDMD-mediated pyroptosis. Inflammation. (2025) 48:181–98. doi: 10.1007/s10753-024-02056-9, PMID: 38773023

[13] Boerma M Azimzadeh O Pasinetti N Monceau V. Editorial: Cardiotoxicity induced by radiotherapy and/or chemotherapy after cancer treatment. Front Oncol. (2022) 12:1087928. doi: 10.3389/fonc.2022.1087928, PMID: 36483033 PMC9723441

[14] Ellahham S Khalouf A Elkhazendar M Dababo N Manla Y. An overview of radiation-induced heart disease. Radiat Oncol J. (2022) 40:89–102. doi: 10.3857/roj.2021.00766, PMID: 35796112 PMC9262704

[15] Ma CX Zhao XK Li YD. New therapeutic insights into radiation-induced myocardial fibrosis. Ther Adv Chronic Dis. (2019) 10:2040622319868383. doi: 10.1177/2040622319868383, PMID: 31448071 PMC6689916

[16] Lynch C Pitroda SP Weichselbaum RR. Radiotherapy, immunity, and immune checkpoint inhibitors. Lancet Oncol. (2024) 25:e352–62. doi: 10.1016/S1470-2045(24)00075-5, PMID: 39089313

[17] Atkins KM Bitterman DS Chaunzwa TL Kozono DE Baldini EH Aerts H. Mean heart dose is an inadequate surrogate for left anterior descending coronary artery dose and the risk of major adverse cardiac events in lung cancer radiation therapy. Int J Radiat Oncol Biol Phys. (2021) 110:1473–9. doi: 10.1016/j.ijrobp.2021.03.005, PMID: 33713743

[18] Borkenhagen JF Bergom C Rapp CT Klawikowski SJ Rein LE Gore EM. Dosimetric predictors of cardiotoxicity in thoracic radiotherapy for lung cancer. Clin Lung Cancer. (2019) 20:435–41. doi: 10.1016/j.cllc.2019.05.014, PMID: 31303452

[19] Brown KN Hussain K Richards JR. Radiation-induced coronary artery disease. In: StatPearls. StatPearls Publishing LLC, Treasure Island (FL (2025). 30725633

[20] Raghunathan D Khilji MI Hassan SA Yusuf SW. Radiation-induced cardiovascular disease. Curr Atheroscler Rep. (2017) 19:22. doi: 10.1007/s11883-017-0658-x, PMID: 28315200

[21] Wang K Chen Y Li Y. Evaluating concordance and clinical utility of ctDNA profiling in advanced biliary tract cancer. J Hepatol. (2025) 82:e320–1. doi: 10.1016/j.jhep.2024.11.014, PMID: 39551391

[22] Yu Z Xu C Song B Zhang S Chen C Li C. Tissue fibrosis induced by radiotherapy: current understanding of the molecular mechanisms, diagnosis and therapeutic advances. J Transl Med. (2023) 21:708. doi: 10.1186/s12967-023-04554-0, PMID: 37814303 PMC10563272

[23] Park J Choi J Cho I Sheen YY. Radiotherapy-induced oxidative stress and fibrosis in breast cancer are suppressed by vactosertib, a novel, orally bioavailable TGF-β/ALK5 inhibitor. Sci Rep. (2022) 12:16104. doi: 10.1038/s41598-022-20050-9, PMID: 36167880 PMC9515166

[24] Chia ZJ Cao YN Little PJ Kamato D. Transforming growth factor-β receptors: versatile mechanisms of ligand activation. Acta Pharmacol Sin. (2024) 45:1337–48. doi: 10.1038/s41401-024-01235-6, PMID: 38351317 PMC11192764

[25] Deng Z Fan T Xiao C Tian H Zheng Y Li C. TGF-β signaling in health, disease, and therapeutics. Signal Transduct Target Ther. (2024) 9:61. doi: 10.1038/s41392-024-01764-w, PMID: 38514615 PMC10958066

[26] Tsuchida K Zhu Y Siva S Dunn SR Sharma K. Role of Smad4 on TGF-beta-induced extracellular matrix stimulation in mesangial cells. Kidney Int. (2003) 63:2000–9. doi: 10.1046/j.1523-1755.2003.00009.x, PMID: 12753287

[27] Chen Y Blom IE Sa S Goldschmeding R Abraham DJ Leask A. CTGF expression in mesangial cells: involvement of SMADs, MAP kinase, and PKC. Kidney Int. (2002) 62:1149–59. doi: 10.1111/j.1523-1755.2002.kid567.x, PMID: 12234285

[28] Lee JH Sánchez-Rivera FJ He L Basnet H Chen FX Spina E. TGF-β and RAS jointly unmask primed enhancers to drive metastasis. Cell. (2024) 187:6182–6199.e6129. doi: 10.1016/j.cell.2024.08.014, PMID: 39243762 PMC12035776

[29] Derynck R Zhang YE. Smad-dependent and Smad-independent pathways in TGF-beta family signalling. Nature. (2003) 425:577–84. doi: 10.1038/nature02006, PMID: 14534577

[30] Kenney LB Ames B Michaud AL Williams DN Manley PE Ullrich NJ. The management of childhood cancer survivors at risk for stroke: A Delphi survey of regional experts. Pediatr Blood Cancer. (2018) 65:e27394. doi: 10.1002/pbc.27394, PMID: 30094926

[31] Van der Vorst A Lambrecht M Van Aelst L Verhoeven J Jacobs J Baten A. Radiation-induced heart disease in breast cancer patients: a narrative review of epidemiology, risk factors, radiotherapy parameters, and prevention. Strahlenther Onkol. (2025) 201:368–82. doi: 10.1007/s00066-024-02362-z, PMID: 39976674

[32] Hoit BD. Pathophysiology of the pericardium. Prog Cardiovasc Dis. (2017) 59:341–8. doi: 10.1016/j.pcad.2016.11.001, PMID: 27916673

[33] Arsenian MA. Cardiovascular sequelae of therapeutic thoracic radiation. Prog Cardiovasc Dis. (1991) 33:299–311. doi: 10.1016/0033-0620(91)90022-E, PMID: 2003146

[34] Ell P Martin JM Cehic DA Ngo DTM Sverdlov AL. Cardiotoxicity of radiation therapy: mechanisms, management, and mitigation. Curr Treat Options Oncol. (2021) 22:70. doi: 10.1007/s11864-021-00868-7, PMID: 34110500

[35] Wei X Liu HH Tucker SL Wang S Mohan R Cox JD. Risk factors for pericardial effusion in inoperable esophageal cancer patients treated with definitive chemoradiation therapy. Int J Radiat Oncol Biol Phys. (2008) 70:707–14. doi: 10.1016/j.ijrobp.2007.10.056, PMID: 18191334

[36] Appleton C Gillam L Koulogiannis K. Cardiac tamponade. Cardiol Clin. (2017) 35:525–37. doi: 10.1016/j.ccl.2017.07.006, PMID: 29025544

[37] Yusuf SW Hassan SA Mouhayar E Negi SI Banchs J O’Gara PT. Pericardial disease: a clinical review. Expert Rev Cardiovasc Ther. (2016) 14:525–39. doi: 10.1586/14779072.2016.1134317, PMID: 26691443

[38] Hu Y Wang K Chen Y Jin Y Guo Q Tang H. Causal relationship between immune cell phenotypes and risk of biliary tract cancer: evidence from Mendelian randomization analysis. Front Immunol. (2024) 15:1430551. doi: 10.3389/fimmu.2024.1430551, PMID: 39050844 PMC11266158

[39] Kucharska W Negrusz-Kawecka M Gromkowska M. Cardiotoxicity of oncological treatment in children. Adv Clin Exp Med. (2012) 21:281–8., PMID: 23214190

[40] Abe JI Allen BG Beyer AM Lewandowski D Mapuskar KA Subramanian V. Radiation-induced macrovessel/microvessel disease. Arterioscler Thromb Vasc Biol. (2024) 44:2407–15. doi: 10.1161/ATVBAHA.124.319866, PMID: 39445428 PMC11842029

[41] Ye S Wang K Li J. Letter to the editor for the article “Impact of postoperative complications on gastric cancer survival. Surgery. (2025) 180:109007. doi: 10.1016/j.surg.2024.109007, PMID: 39730263

[42] Zhao Y Chen X Huang Y Zhang Z Wang K Zou D. Transcriptomic insights into hub genes, immune infiltration, and candidate drugs in erosive esophagitis. J Inflammation Res. (2024) 17:7745–60. doi: 10.2147/JIR.S479032, PMID: 39494202 PMC11529285

[43] Gkantaifi A Papadopoulos C Spyropoulou D Toumpourleka M Iliadis G Tsoukalas N. Evaluation of the irradiated volume of the heart and cardiac substructures after left breast radiotherapy. Anticancer Res. (2020) 40:3003–9. doi: 10.21873/anticanres.14281, PMID: 32366455

[44] Gyenes G Gagliardi G Lax I Fornander T Rutqvist LE. Evaluation of irradiated heart volumes in stage I breast cancer patients treated with postoperative adjuvant radiotherapy. J Clin Oncol. (1997) 15:1348–53. doi: 10.1200/JCO.1997.15.4.1348, PMID: 9193326

[45] Tjessem KH Bosse G Fosså K Reinertsen KV Fosså SD Johansen S. Coronary calcium score in 12-year breast cancer survivors after adjuvant radiotherapy with low to moderate heart exposure - Relationship to cardiac radiation dose and cardiovascular risk factors. Radiother Oncol. (2015) 114:328–34. doi: 10.1016/j.radonc.2015.01.006, PMID: 25600105

[46] Darby SC Ewertz M McGale P Bennet AM Blom-Goldman U Brønnum D. Risk of ischemic heart disease in women after radiotherapy for breast cancer. N Engl J Med. (2013) 368:987–98. doi: 10.1056/NEJMoa1209825, PMID: 23484825

[47] Liu LK Ouyang W Zhao X Su Sh F Yang Y Ding WJ. Pathogenesis and prevention of radiation-induced myocardial fibrosis. Asian Pac J Cancer Prev. (2017) 18:583–7. doi: 10.22034/APJCP.2017.18.3.583, PMID: 28440606 PMC5464468

[48] Heidenreich PA Hancock SL Lee BK Mariscal CS Schnittger I. Asymptomatic cardiac disease following mediastinal irradiation. J Am Coll Cardiol. (2003) 42:743–9. doi: 10.1016/S0735-1097(03)00759-9, PMID: 12932613

[49] Saiki H Moulay G Guenzel AJ Liu W Decklever TD Classic KL. Experimental cardiac radiation exposure induces ventricular diastolic dysfunction with preserved ejection fraction. Am J Physiol Heart Circ Physiol. (2017) 313:H392–h407. doi: 10.1152/ajpheart.00124.2017, PMID: 28550173 PMC5582918

[50] Chang HM Okwuosa TM Scarabelli T Moudgil R Yeh ETH. Cardiovascular complications of cancer therapy: best practices in diagnosis, prevention, and management: part 2. J Am Coll Cardiol. (2017) 70:2552–65. doi: 10.1016/j.jacc.2017.09.1095, PMID: 29145955 PMC5825188

[51] Mulrooney DA Yeazel MW Kawashima T Mertens AC Mitby P Stovall M. Cardiac outcomes in a cohort of adult survivors of childhood and adolescent cancer: retrospective analysis of the Childhood Cancer Survivor Study cohort. Bmj. (2009) 339:b4606. doi: 10.1136/bmj.b4606, PMID: 19996459 PMC3266843

[52] Donnellan E Masri A Johnston DR Pettersson GB Rodriguez LL Popovic ZB. Long-term outcomes of patients with mediastinal radiation-associated severe aortic stenosis and subsequent surgical aortic valve replacement: A matched cohort study. J Am Heart Assoc. (2017) 6:e005396. doi: 10.1161/JAHA.116.005396, PMID: 28476874 PMC5524090

[53] Bijl JM Roos MM van Leeuwen-Segarceanu EM Vos JM Bos WW Biesma DH. Assessment of valvular disorders in survivors of hodgkin’s lymphoma treated by mediastinal radiotherapy ± Chemotherapy. Am J Cardiol. (2016) 117:691–6. doi: 10.1016/j.amjcard.2015.11.027, PMID: 26772441

[54] Országhová Z Mego M Chovanec M. Long-term cognitive dysfunction in cancer survivors. Front Mol Biosci. (2021) 8:770413. doi: 10.3389/fmolb.2021.770413, PMID: 34970595 PMC8713760

[55] Chen L Cai X Shao L Wang Y Hong L Zhan Y. Sirtuin 2 exerts regulatory functions on radiation-induced myocardial fibrosis in mice by mediating H3K27 acetylation of galectin-3 promoter. Acta Cardiol Sin. (2024) 40:214–24. doi: 10.6515/ACS.202403_40(2).20231026B, PMID: 38532816 PMC10961639

[56] Erbay MI Manubolu VS Stein-Merlob AF Ferencik M Mamas MA Lopez-Mattei J. Integration and potential applications of cardiovascular computed tomography in cardio-oncology. Curr Cardiol Rep. (2025) 27:51. doi: 10.1007/s11886-025-02206-x, PMID: 39932640 PMC11814013

[57] Cutter DJ Schaapveld M Darby SC Hauptmann M van Nimwegen FA Krol AD. Risk of valvular heart disease after treatment for Hodgkin lymphoma. J Natl Cancer Inst. (2015) 107:djv008. doi: 10.1093/jnci/djv008, PMID: 25713164 PMC4394894

[58] Wethal T Lund MB Edvardsen T Fosså SD Pripp AH Holte H. Valvular dysfunction and left ventricular changes in Hodgkin’s lymphoma survivors. A longitudinal study. Br J Cancer. (2009) 101:575–81. doi: 10.1038/sj.bjc.6605191, PMID: 19623176 PMC2736805

[59] van Nimwegen FA Schaapveld M Janus CP Krol AD Petersen EJ Raemaekers JM. Cardiovascular disease after Hodgkin lymphoma treatment: 40-year disease risk. JAMA Intern Med. (2015) 175:1007–17. doi: 10.1001/jamainternmed.2015.1180, PMID: 25915855

[60] Jang BS Cha MJ Kim HJ Oh S Wu HG Kim E. Heart substructural dosimetric parameters and risk of cardiac events after definitive chemoradiotherapy for stage III non-small cell lung cancer. Radiother Oncol. (2020) 152:126–32. doi: 10.1016/j.radonc.2020.09.050, PMID: 33058951

[61] Du S Zhou L Alexander GS Park K Yang L Wang N. PD-1 modulates radiation-induced cardiac toxicity through cytotoxic T lymphocytes. J Thorac Oncol. (2018) 13:510–20. doi: 10.1016/j.jtho.2017.12.002, PMID: 29247829 PMC9335897

[62] Venkatesulu BP Mahadevan LS Aliru ML Yang X Bodd MH Singh PK. Radiation-induced endothelial vascular injury: A review of possible mechanisms. JACC Basic Transl Sci. (2018) 3:563–72. doi: 10.1016/j.jacbts.2018.01.014, PMID: 30175280 PMC6115704

[63] Wang B Wang H Zhang M Ji R Wei J Xin Y. Radiation-induced myocardial fibrosis: Mechanisms underlying its pathogenesis and therapeutic strategies. J Cell Mol Med. (2020) 24:7717–29. doi: 10.1111/jcmm.15479, PMID: 32536032 PMC7348163

[64] Cuomo JR Sharma GK Conger PD Weintraub NL. Novel concepts in radiation-induced cardiovascular disease. World J Cardiol. (2016) 8:504–19. doi: 10.4330/wjc.v8.i9.504, PMID: 27721934 PMC5039353

[65] Schultz-Hector S Trott KR. Radiation-induced cardiovascular diseases: is the epidemiologic evidence compatible with the radiobiologic data? Int J Radiat Oncol Biol Phys. (2007) 67:10–8. doi: 10.1016/j.ijrobp.2006.08.071, PMID: 17189062

[66] Aleman BM van den Belt-Dusebout AW De Bruin ML van ‘t Veer MB Baaijens MH de Boer JP. Late cardiotoxicity after treatment for Hodgkin lymphoma. Blood. (2007) 109:1878–86. doi: 10.1182/blood-2006-07-034405, PMID: 17119114

[67] Cella L Liuzzi R Conson M Torre G Caterino M De Rosa N. Dosimetric predictors of asymptomatic heart valvular dysfunction following mediastinal irradiation for Hodgkin’s lymphoma. Radiother Oncol. (2011) 101:316–21. doi: 10.1016/j.radonc.2011.08.040, PMID: 21925755

[68] Koutroumpakis E Deswal A Yusuf SW Abe JI Nead KT Potter AS. Radiation-induced cardiovascular disease: mechanisms, prevention, and treatment. Curr Oncol Rep. (2022) 24:543–53. doi: 10.1007/s11912-022-01238-8, PMID: 35192118

[69] Picano E Pierard L Peteiro J Djordjevic-Dikic A Sade LE Cortigiani L. The clinical use of stress echocardiography in chronic coronary syndromes and beyond coronary artery disease: a clinical consensus statement from the European Association of Cardiovascular Imaging of the ESC. Eur Heart J Cardiovasc Imaging. (2024) 25:e65–90. doi: 10.1093/ehjci/jead250, PMID: 37798126

[70] Galli E Soliman-Aboumarie H Gargani L Szymański P Gimelli A Petersen SE. EACVI survey on radiation exposure in interventional echocardiography. Eur Heart J Cardiovasc Imaging. (2024) 25:727–34. doi: 10.1093/ehjci/jeae086, PMID: 38635738 PMC11139519

[71] Plana JC Galderisi M Barac A Ewer MS Ky B Scherrer-Crosbie M. Expert consensus for multimodality imaging evaluation of adult patients during and after cancer therapy: a report from the American Society of Echocardiography and the European Association of Cardiovascular Imaging. J Am Soc Echocardiogr. (2014) 27:911–39. doi: 10.1016/j.echo.2014.07.012, PMID: 25172399

[72] Lancellotti P Nkomo VT Badano LP Bergler-Klein J Bogaert J Davin L. Expert consensus for multi-modality imaging evaluation of cardiovascular complications of radiotherapy in adults: a report from the European Association of Cardiovascular Imaging and the American Society of Echocardiography. Eur Heart J Cardiovasc Imaging. (2013) 14:721–40. doi: 10.1093/ehjci/jet123, PMID: 23847385

[73] Clasen SC Wald JW. Left ventricular dysfunction and chemotherapeutic agents. Curr Cardiol Rep. (2018) 20:20. doi: 10.1007/s11886-018-0967-x, PMID: 29520629

[74] Davis M Witteles RM. Radiation-induced heart disease: an under-recognized entity? Curr Treat Options Cardiovasc Med. (2014) 16:317. doi: 10.1007/s11936-014-0317-2, PMID: 24756471

[75] Amsterdam EA Wenger NK Brindis RG Casey DE Jr. Ganiats TG Holmes DR Jr.. 2014 AHA/ACC guideline for the management of patients with non-ST-elevation acute coronary syndromes: a report of the American College of Cardiology/American Heart Association Task Force on practice guidelines. J Am Coll Cardiol. (2014) 64:e139–228. doi: 10.1016/j.jacc.2014.09.017, PMID: 25260718

[76] Erven K Florian A Slagmolen P Sweldens C Jurcut R Wildiers H. Subclinical cardiotoxicity detected by strain rate imaging up to 14 months after breast radiation therapy. Int J Radiat Oncol Biol Phys. (2013) 85:1172–8. doi: 10.1016/j.ijrobp.2012.09.022, PMID: 23149005

[77] Nellessen U Zingel M Hecker H Bahnsen J Borschke D. Effects of radiation therapy on myocardial cell integrity and pump function: which role for cardiac biomarkers? Chemotherapy. (2010) 56:147–52. doi: 10.1159/000313528, PMID: 20407242

[78] Omran F Kyrou I Osman F Lim VG Randeva HS Chatha K. Cardiovascular biomarkers: lessons of the past and prospects for the future. Int J Mol Sci. (2022) 23:5680. doi: 10.3390/ijms23105680, PMID: 35628490 PMC9143441

[79] Walls GM Hill N McMahon M Kearney B McCann C McKavanagh P. Baseline cardiac parameters as biomarkers of radiation cardiotoxicity in lung cancer: an NI-HEART analysis. JACC Cardio Oncol. (2024) 6:529–40. doi: 10.1016/j.jaccao.2024.05.009, PMID: 39239328 PMC11372030

[80] Roy DC Wang TF Mallick R Carrier M Mollanji E Liu P. Growth differentiation factor-15, high-sensitivity cardiac troponin T, and N-terminal pro-B-type natriuretic peptide for predicting risk of venous thromboembolism in ambulatory cancer patients receiving chemotherapy. Thromb Haemost. (2022) 122:1169–76. doi: 10.1055/a-1792-7720, PMID: 35263789

[81] Palaskas N Patel A Yusuf SW. Radiation and cardiovascular disease. Ann Transl Med. (2019) 7:S371. doi: 10.21037/atm.2019.08.107, PMID: 32016089 PMC6976451

[82] Filippi AR Meregalli S A DIR Levis M Ciammella P Buglione M. Fondazione Italiana Linfomi (FIL) expert consensus on the use of intensity-modulated and image-guided radiotherapy for Hodgkin’s lymphoma involving the mediastinum. Radiat Oncol. (2020) 15:62. doi: 10.1186/s13014-020-01504-8, PMID: 32164700 PMC7066773

[83] Tomaszewski JM Crook S Wan K Scott L Foroudi F. A case study evaluating deep inspiration breath-hold and intensity-modulated radiotherapy to minimise long-term toxicity in a young patient with bulky mediastinal Hodgkin lymphoma. J Med Radiat Sci. (2017) 64:69–75. doi: 10.1002/jmrs.219, PMID: 28188697 PMC5355368

[84] Lin SH Hobbs BP Verma V Tidwell RS Smith GL Lei X. Randomized phase IIB trial of proton beam therapy versus intensity-modulated radiation therapy for locally advanced esophageal cancer. J Clin Oncol. (2020) 38:1569–79. doi: 10.1200/JCO.19.02503, PMID: 32160096 PMC7213588

[85] Favaudon V Caplier L Monceau V Pouzoulet F Sayarath M Fouillade C. Ultrahigh dose-rate FLASH irradiation increases the differential response between normal and tumor tissue in mice. Sci Transl Med. (2014) 6:245ra293. doi: 10.1126/scitranslmed.3008973, PMID: 25031268

[86] McGarrigle JM Long KR Prezado Y. The FLASH effect-an evaluation of preclinical studies of ultra-high dose rate radiotherapy. Front Oncol. (2024) 14:1340190. doi: 10.3389/fonc.2024.1340190, PMID: 38711846 PMC11071325

[87] Kim K Kim MM Skoufos G Diffenderfer ES Motlagh SAO Kokkorakis M. FLASH proton radiation therapy mitigates inflammatory and fibrotic pathways and preserves cardiac function in a preclinical mouse model of radiation-induced heart disease. Int J Radiat Oncol Biol Phys. (2024) 119:1234–47. doi: 10.1016/j.ijrobp.2024.01.224, PMID: 38364948 PMC11209795

[88] Vozenin MC Bourhis J Durante M. Towards clinical translation of FLASH radiotherapy. Nat Rev Clin Oncol. (2022) 19:791–803. doi: 10.1038/s41571-022-00697-z, PMID: 36303024

[89] Schiffer W Pedersen LN Lui M Bergom C Mitchell JD. Advances in screening for radiation-associated cardiotoxicity in cancer patients. Curr Cardiol Rep. (2023) 25:1589–600. doi: 10.1007/s11886-023-01971-x, PMID: 37796395 PMC10682284

[90] Zhu L Wang Y Zhao S Lu M. Detection of myocardial fibrosis: Where we stand. Front Cardiovasc Med. (2022) 9:926378. doi: 10.3389/fcvm.2022.926378, PMID: 36247487 PMC9557071

[91] Lancellotti P Price S Edvardsen T Cosyns B Neskovic AN Dulgheru R. The use of echocardiography in acute cardiovascular care: recommendations of the European Association of Cardiovascular Imaging and the Acute Cardiovascular Care Association. Eur Heart J Cardiovasc Imaging. (2015) 16:119–46. doi: 10.1093/ehjci/jeu210, PMID: 25378470

[92] Greulich S Mayr A Kitterer D Latus J Henes J Steubing H. T1 and T2 mapping for evaluation of myocardial involvement in patients with ANCA-associated vasculitides. J Cardiovasc Magn Reson. (2017) 19:6. doi: 10.1186/s12968-016-0315-5, PMID: 28077133 PMC5225624

[93] Radunski UK Lund GK Säring D Bohnen S Stehning C Schnackenburg B. T1 and T2 mapping cardiovascular magnetic resonance imaging techniques reveal unapparent myocardial injury in patients with myocarditis. Clin Res Cardiol. (2017) 106:10–7. doi: 10.1007/s00392-016-1018-5, PMID: 27388331

[94] Chang M Suh J Kirtani V Dobrescu A Haas J Zeldis S. Coronary calcium scanning in patients after adjuvant radiation for early breast cancer and ductal carcinoma. *In situ* . Front Oncol. (2013) 3:253. doi: 10.3389/fonc.2013.00253, PMID: 24093087 PMC3782706

[95] Huang JY Huang CK Yen RF Chien KL Wu YW. Diagnostic effect of attenuation correction in myocardial perfusion imaging in different coronary arteries: A systematic review and meta-analysis. Front Cardiovasc Med. (2021) 8:756060. doi: 10.3389/fcvm.2021.756060, PMID: 34712715 PMC8545877

[96] D’Errico MP Petruzzelli MF Gianicolo EA Grimaldi L Loliva F Tramacere F. Kinetics of B-type natriuretic peptide plasma levels in patients with left-sided breast cancer treated with radiation therapy: Results after one-year follow-up. Int J Radiat Biol. (2015) 91:804–9. doi: 10.3109/09553002.2015.1027421, PMID: 25955228

[97] D’Errico MP Grimaldi L Petruzzelli MF Gianicolo EA Tramacere F Monetti A. N-terminal pro-B-type natriuretic peptide plasma levels as a potential biomarker for cardiac damage after radiotherapy in patients with left-sided breast cancer. Int J Radiat Oncol Biol Phys. (2012) 82:e239–246. doi: 10.1016/j.ijrobp.2011.03.058, PMID: 21640499

[98] Zaborowska-Szmit M Szmit S Olszyna-Serementa M Badurak P Zajda K Janowicz-Żebrowska A. Beta blockers with statins may decrease all-cause mortality in patients with cardiovascular diseases and locally advanced unresectable non-small-cell lung cancer after chemoradiotherapy. Cancers (Basel). (2023) 15:1277. doi: 10.3390/cancers15041277, PMID: 36831618 PMC9954027

[99] Yusuf SW Sami S Daher IN. Radiation-induced heart disease: a clinical update. Cardiol Res Pract. (2011) 2011:317659. doi: 10.4061/2011/317659, PMID: 21403872 PMC3051159

[100] Totzeck M Mincu RI Heusch G Rassaf T. Heart failure from cancer therapy: can we prevent it? ESC Heart Fail. (2019) 6:856–62. doi: 10.1002/ehf2.12493, PMID: 31297946 PMC6676296

[101] Menezes KM Wang H Hada M Saganti PB. Radiation matters of the heart: A mini review. Front Cardiovasc Med. (2018) 5:83. doi: 10.3389/fcvm.2018.00083, PMID: 30038908 PMC6046516

[102] Christersdottir T Pirault J Gisterå A Bergman O Gallina AL Baumgartner R. Prevention of radiotherapy-induced arterial inflammation by interleukin-1 blockade. Eur Heart J. (2019) 40:2495–503. doi: 10.1093/eurheartj/ehz206, PMID: 31081038 PMC6685328

